# Prenatal exposure to predator odor alters placental glucocorticoid metabolism and affects fetal growth in Brandt's voles (*Lasiopodomys brandtii*)

**DOI:** 10.1093/cz/zoaf043

**Published:** 2025-07-28

**Authors:** Chen Gu, Yang Yu, Yuqing Zhang, Fengping Yang, Shengmei Yang, Baofa Yin, Wanhong Wei

**Affiliations:** Department of Animal Behavior, College of Bioscience and Biotechnology, Yangzhou University, Wenhui East Road No.48, Yangzhou, Jiangsu 225009, China; Department of Animal Behavior, College of Bioscience and Biotechnology, Yangzhou University, Wenhui East Road No.48, Yangzhou, Jiangsu 225009, China; Department of Animal Behavior, College of Bioscience and Biotechnology, Yangzhou University, Wenhui East Road No.48, Yangzhou, Jiangsu 225009, China; Department of Animal Behavior, College of Bioscience and Biotechnology, Yangzhou University, Wenhui East Road No.48, Yangzhou, Jiangsu 225009, China; Department of Animal Behavior, College of Bioscience and Biotechnology, Yangzhou University, Wenhui East Road No.48, Yangzhou, Jiangsu 225009, China; Department of Animal Behavior, College of Bioscience and Biotechnology, Yangzhou University, Wenhui East Road No.48, Yangzhou, Jiangsu 225009, China; Department of Animal Behavior, College of Bioscience and Biotechnology, Yangzhou University, Wenhui East Road No.48, Yangzhou, Jiangsu 225009, China

**Keywords:** Brandt's voles, fetal development, glucocorticoid metabolism, predator odor, prenatal stress

## Abstract

Environmental stressors encountered during pregnancy can exert profound effects on fetal development and program long-term physiological outcomes in offspring. Among these, ecologically relevant stressors such as predation risk remain understudied despite their potential to disrupt maternal physiology and placental function. In this study, we investigated how prenatal exposure to predator odor influences placental glucocorticoid metabolism and fetal growth in Brandt's voles (*Lasiopodomys brandtii*). Pregnant voles were exposed to predator odor to simulate prenatal predator stress, allowing us to assess its effects on fetal growth, maternal and fetal corticosterone levels, and the expression of key enzymes and transport proteins involved in glucocorticoid regulation. The results revealed that prenatal exposure to predator odor significantly reduced fetal weight. While maternal and fetal serum corticosterone levels increased, placental corticosterone levels remained unchanged. Additionally, our study observed significant increases in the expression of placental P-glycoprotein and 11β-hydroxysteroid dehydrogenase 1, both of which are crucial for maintaining placental glucocorticoid metabolic homeostasis. However, 11β-hydroxysteroid dehydrogenase 2 levels were unaffected. In the fetal brain, corticotropin-releasing hormone expression in the hypothalamus showed a downward trend, whereas glucocorticoid receptor expression in the hippocampus remained unchanged, indicating a disruption in the development of the hypothalamic-pituitary-adrenal axis. These findings suggest that prenatal exposure to predator odor alters placental glucocorticoid metabolism, leading to increased fetal corticosterone exposure and potentially impairing fetal development.

## Graphical Abstract

**Graphical Abstract:**
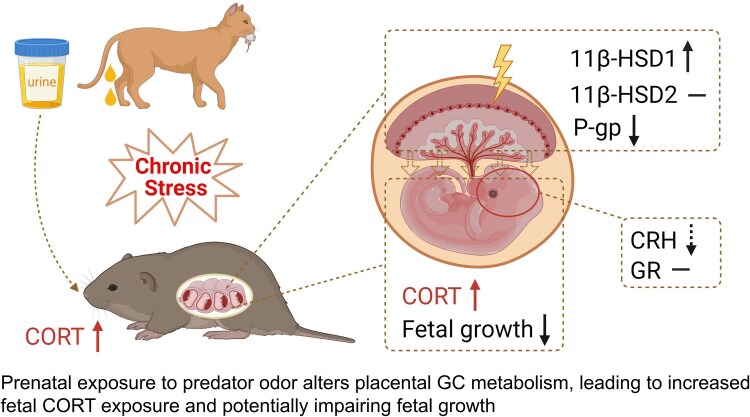

Odor cues can convey critical information regarding the presence and activity of predators in the environment, enabling prey to take appropriate actions to reduce the risk of predation (Kats and Dill 1998; Apfelbach et al. 2005; Takahashi et al. 2005). Upon detecting predator odors, prey often exhibit heightened vigilance, decreased foraging activity, and altered habitat use, prioritizing safety over other essential behaviors (Kats and Dill 1998; Adduci et al. 2021; Maestas-Olguin et al. 2021; Chen et al. 2022). These adaptive behavioral shifts, though enhancing survival chances, are energetically costly and often come at the expense of growth or reproductive output, resulting in trade-offs that can diminish body condition or delay reproduction (Creel and Christianson 2008; Clinchy et al. 2013; Zanette and Clinchy 2019). One key mechanism underlying these responses is the activation of the hypothalamic-pituitary-adrenal (HPA) axis, particularly through the secretion of glucocorticoids (GCs), which mediate both behavioral and physiological adaptations in prey (Harris and Carr 2016). Acute exposure to predator cues triggers a rapid surge in GC secretion, leading to immediate physiological and behavioral changes to promote survival (Bennett et al. 2016; Brachetta et al. 2020). This acute response is typically followed by negative feedback mechanisms that terminate GC secretion once the threat has passed, allowing the organism to restore homeostasis (Newman et al. 2013; Elvidge et al. 2014). However, long-term exposure to predator cues can prolong HPA axis activation, resulting in persistently elevated GC levels that can impair normal physiological and reproductive functions (Campos et al. 2013).

Olfactory information about predation risk can also be transmitted intergenerationally from mother to offspring, significantly influencing offspring growth, development, and behavioral phenotype well into adulthood. For pregnant individuals, detecting predator odors and responding with increased secretion of catecholamines and GCs, along with adaptive behavioral changes, serves as a strategy to enhance both immediate survival and long-term reproductive success (Takahashi 2014; Harris and Carr 2016). However, chronic maternal stress can lead to sustained elevations in circulating corticosterone (CORT), thereby increasing fetal exposure to GCs during critical stages of development (McGowan and Matthews 2018). This intergenerational transfer of predator-associated stress signals effectively “prepares” offspring for a high-risk environment, priming them to better cope with predation threats later in life. For example, bank vole (*Myodes glareolus*) pups exposed to maternal predator odor exhibited increased risk-taking behavior when exposed to either predator odor or alarm pheromone (Sievert et al. 2020). Similar patterns appear in aquatic species such as the cichlid fish (*Neolamprologus pulcher*), where maternal exposure to predator cues modifies egg composition and produces offspring with faster escape responses and enhanced predator avoidance (Sharda et al. 2021). Nevertheless, if the anticipated predation risks fail to materialize, such stress-induced phenotypes can become maladaptive, resulting in a mismatch between offspring traits and actual environmental demands, ultimately reducing fitness (Marshall and Uller 2007; Sheriff and Love 2013; St-Cyr and McGowan 2018).

One of the potential mechanisms underlying these intergenerational effects involves altered placental GC metabolism. When the regulation of GCs by the placenta is disrupted, the fetus may be exposed to excessive levels of maternal GCs, which can inhibit fetal cell proliferation and profoundly impair growth and development (Konstantakou et al. 2017). Under normal conditions, the placenta relies on 11β-hydroxysteroid dehydrogenases (11β-HSDs) to modulate GC metabolism. Two key isoforms, 11β-HSD1 and 11β-HSD2, play distinct yet complementary roles in this process: 11β-HSD2 primarily inactivates CORT, thereby shielding the fetus from excessive maternal GCs, while 11β-HSD1 regenerates active CORT in peripheral tissue (Cottrell et al. 2014). In addition, the ATP-dependent transporter P-glycoprotein (P-gp) contributes to this protective mechanism by limiting GC transfer across the placenta, thereby further reducing fetal GC exposure (Ceckova-Novotna et al. 2006). Together, 11β-HSDs and P-gp form a coordinated defense system that safeguards fetal development by tightly regulating maternal GC influence (Karssen et al. 2001; Harris and Seckl 2011). Studies on maternal stress in rodent models have shown that various environmental stressors can adversely affect the expression of key enzymes and proteins involved in GC metabolism in the placenta (Kapoor et al. 2008; Conradt et al. 2013). For example, maternal psychological stress or chronic inflammatory conditions can disrupt the normal functioning of 11β-HSDs and P-gp. This disruption may result in increased fetal exposure to active GCs, which has been linked to impaired fetal development and long-term health consequences (Drake et al. 2007; O’donnell et al. 2009; Wieczorek et al. 2019). These proteins thus serve a crucial function by limiting the transfer of maternal GCs. Despite growing knowledge of how maternal stress affects placental function, the specific impact of ecological stressors, such as predator odor, on these processes remains poorly understood. Direct measurement of fetal CORT levels during maternal exposure to predator cues is technically challenging. However, changes in the expression of placental 11β-HSDs and P-gp may serve as indirect indicators of altered fetal GC exposure under such stress conditions. These placental mechanisms are likely to mediate how maternal perception of predation risk influences fetal development. To understand the consequences of predator-induced stress during pregnancy, further research is needed to determine how these protective systems respond to such stressors.

The Brandt's vole (*Lasiopodomys brandtii*) is a key rodent species in the steppe grasslands of Inner Mongolia, essential for local biodiversity (Samjaa et al. 2000; Li et al. 2024). These voles exhibit seasonal breeding, with peak reproductive activity typically observed from March to July. Both sexes reach sexual maturity at ∼40–45 days, and the gestation period lasts between 21 and 23 days. Females can produce up to 6 litters annually, with each litter containing between 2 and 12 pups, averaging about 6.1 pups per litter (Gromov 2023). Regularly exposed to predators such as raptors, felines, canids, weasels, and snakes (Winters et al. 2010), Brandt's voles serve as an ideal model for studying physiological and behavioral adaptations to predation risk. Our previous studies found that these voles can distinguish between predator and non-predator odors (Hegab et al. 2013), and exposure to predator odor triggers notable changes in their defensive behaviors and HPA axis responses (Hegab et al. 2014). Repeated exposure to predator odor during pregnancy also affects the growth and foraging strategies of offspring in Brandt's voles (Gu et al. 2018). However, the mechanisms by which maternal predator stress during pregnancy impacts offspring phenotypes are still largely unknown. In this study, we examined how prenatal exposure to predator odor affects fetal development in Brandt's voles by measuring changes in fetal number and weight, as well as CORT concentrations in maternal, placental, and fetal compartments. We also quantified expression levels of placental 11β-HSD1, 11β-HSD2, and P-gp, which are key regulators of GC metabolism. We hypothesized that repeated predator odor exposure during pregnancy would disrupt the expression of these regulatory proteins, increase fetal exposure to maternal CORT, and consequently impair fetal development.

## Materials and methods

### Experimental animals

Animals were offspring of Brandt's voles (*Lasiopodomys brandtii*) trapped in Inner Mongolian grasslands in May 2010 and raised at the Department of Animal Behavior, Yangzhou University in Yangzhou. Approximately 70 breeding pairs of voles are maintained year-round, and inbreeding is minimized by controlling breeding pairs, monitoring lineage, and rejuvenating either with a wild population or with another laboratory every 2 years. All voles are maintained at a controlled temperature of 23 °C and under a light/dark period of 12 h, with the light period beginning at 07:00 h. They were fed an adequate standard pellet diet (Yizheng Animal Biotechnology Co., LTD, Yangzhou, China) and provided with distilled water throughout the experimental period. Cages are cleaned and bedding is replaced on a regular schedule twice weekly, balancing hygiene with the need to retain familiar odors to prevent undue stress.

### Odor sources

The cat urine used in this study was collected from an adult male domestic cat (*Felis catus*) caught on the Wenhui campus of Yangzhou University. The rabbit urine was collected from an adult male domestic rabbit (*Oryctolagus curiculus*) bought at a pet shop in Yangzhou. Urine samples were collected daily by placing clean trays under the cat and rabbit cages, then stored at −20 °C until a sufficient volume was obtained. A fine metal mesh positioned between the cages and trays prevents animal feces from contaminating the samples. To reduce variability between collections, samples from different batches are thawed once, thoroughly mixed, and aliquoted. Prior to odor exposure, these aliquots are pre-thawed, brought to room temperature, and diluted fivefold with distilled water for subsequent use. The diluted cat and rabbit urine was used as sources of predator and non-predator odors, respectively, while distilled water served as the control. We have demonstrated that upon acute exposure to cat urine odor diluted 5-fold, Brandt's voles exhibited significantly heightened anti-predation behavior compared to exposure to either diluted rabbit urine or distilled water (Yu, 2024). Moreover, prolonged exposure to cat odor affects both their behavior and physiological responses, as well as those of their offspring, indicating a clear intergenerational effect (Gu et al. 2018; Wu et al. 2020, 2023).

### Experimental procedures

**Figure 1:**
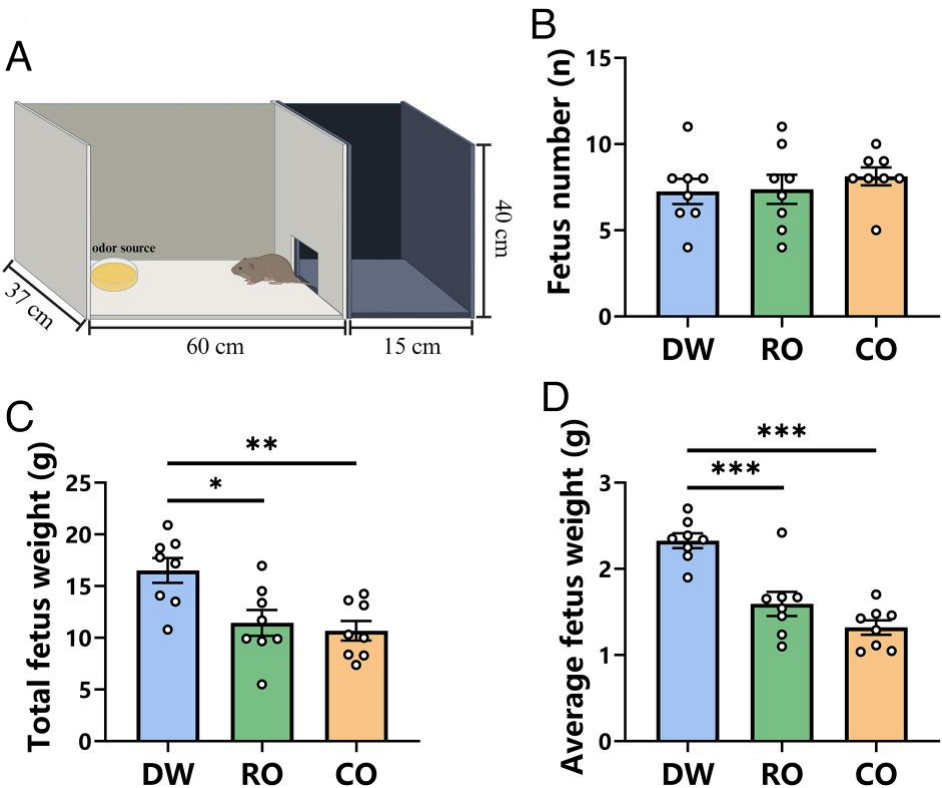
Effect of prenatal exposure to different odors on fetus number and fetus weight of Brandt's voles. (A) Schematic diagram of the apparatus dimensions and the manner of odor exposure; (B) fetus number; (C) total fetus weight; (D) average fetus weight. DW, distilled water exposure group, *n* = 8; RO, rabbit odor exposure group, *n* = 8; CO, cat odor exposure group, *n* = 8. Data are mean ± SEM. Significant differences are indicated: *P < 0.05, **P < 0.01, ***P < 0.001.

Twenty-four male Brandt's voles (62 ± 1 g), each with visibly developed testes, were paired with estrous females of similar body weight (50 ± 1 g), identified by the presence of an open, moist vaginal opening. All voles were 14 weeks old and originated from different mothers to minimize genetic relatedness. Each breeding pair was housed individually in a cage (485 × 350 × 200 mm). Females were monitored daily for the presence of a vaginal plug, indicating successful mating, upon which the males were removed. The exposure procedure followed protocols established in previous studies (Gu et al. 2018; Wu et al. 2020, 2023). Briefly, pregnant voles were randomly assigned to 3 groups, with 8 individuals per group. The voles in 3 groups were respectively exposed to petri dishes containing 10 mL of distilled water (DW), diluted rabbit urine (rabbit odor, RO), or diluted cat urine (cat odor, CO) in the test apparatus for one hour (Fig. 1A). Subsequently, all animals were returned to their respective cages. After each exposure trial, the test apparatus was thoroughly cleaned with 70% ethanol, followed by distilled water to remove any residual odors and avoid contamination between trials. These procedures were conducted daily for 18 consecutive days. On the following day, pregnant voles underwent euthanasia via injection with pentobarbital sodium, followed by decapitation for blood collection. The number and weight of fetuses were recorded accordingly. Two placentas were randomly selected from each female vole to measure levels of CORT, P-gp, 11β-HSD1 and 11β-HSD2. Correspondingly, 2 fetuses associated with these placentas were chosen to assess blood CORT levels and the expression of CRH and GR in brain tissue. Selecting 2 placentas and 2 fetuses from each female vole was to reduce biological variability and ensure a manageable sample size.

### Measurements of serum CORT and placental P-gp concentrations

The enzyme-linked immunosorbent assay (ELISA) for serum and tissue was conducted based on the method by Wu et al. (2022), with minor modifications. Briefly, the serum samples were separated from blood using 3,000 rpm centrifugation at 4 °C for 30 min and then stored at −20 °C. 0.1 g sample of placental tissue was added to 1 mL of PBS, fully homogenized, and then centrifuged at 3,000 rpm for 30 min. The supernatant was collected to prepare the placental sample. Serum CORT and placental P-gp levels were determined using an ELISA kit for voles (Shanghai Jianglai Industrial Limited by Share Ltd., Shanghai, China). The relevant testing process was carried out following the instructions. The optical density of each sample was determined at 450 nm using a Metertech microplate reader (BioTek Instruments Co., Ltd., Vermont, USA).

### Measurements of CRH, GR, 11β-HSD1, and 11β-HSD2 expression

The protein expression of CRH, GR, 11β-HSD1 and 11β-HSD2 were measured by Western Blot. Total proteins from the fetal brain or placenta were extracted with RIPA lysis buffer containing a protease inhibitor (Chudnovets et al. 2020), and sample protein concentrations were determined with BCA assays (E112-01/02, Vazyme Biotech Co. Ltd., Nanjing, China). Denatured protein extract was separated using 10% SDS-PAGE gel electrophoresis and transferred to a PVDF membrane. The membrane was incubated with rabbit polyclonal anti-CRH (1:2,000, 10944-1-AP, Proteintech, China), anti-GR (1:2,000, 10944-1-AP, Proteintech, China), anti-11β-HSD1 (1:1,000, 10928-1-AP, Proteintech, China), anti-11β-HSD2 (1:1,000, 14192-1-AP, Proteintech, China), anti-GAPDH (1:2,000, 10494-1-AP, Proteintech, China) or anti-β-actin (1:2,000, bs-0061R, Bioss, China) at 4 °C overnight. Following washing, the membrane was incubated with HRP-conjugated goat anti-rabbit IgG and then visualized using an enhanced ECL kit and a luminescent and fluorescent imaging system (Tanon 5200, Shanghai, China). Western blot images were analyzed using ImageJ software (Schneider et al. 2012). GAPDH (for placental protein blots) and β-actin (for fetal brain blots) were used as internal loading controls to normalize target protein expression. All quantifications were based on band intensity relative to the respective housekeeping protein.

### Data analysis

SPSS statistical analysis software (SPSS 22.0 for Windows) was used to analyze the obtained data statistically. The measurement values from the 2 placentas or 2 fetuses of each female were averaged to generate a single data point, avoiding pseudoreplication. One-way ANOVA was used to analyze data for indices related to placental barrier and fetal development in Brandt's voles, provided that the assumption of normality and the assumption of homogeneity of variance (Levene's test) were met. Further, Tukey's honestly significant difference (HSD) post hoc test was performed if there were differences in the main effects of ANOVA. The Pearson correlation coefficient was used to study the correlation between hormonal and related protein expression. Data in the text are expressed as mean ± standard error (mean ± SEM), with *P* < 0.05 as the criterion for significant differences.

## Results

### The number and weight of the fetuses

Pregnant Brandt's voles were exposed daily to DW, RO, or CO using the odor exposure apparatus shown in Fig. 1A. Fetal number and weight were assessed at the end of the exposure period. The one-way ANOVA results indicated that prenatal exposure to different odors had no significant effect on the number of fetuses in Brandt's voles (*F*_2, 21_ = 0.447, *P* = 0.646, Fig. 1B). However, it significantly impacted both total fetal weight (*F*_2, 21_ = 7.751, *P* = 0.003) and average fetal weight (*F*_2, 21_ = 23.951, *P* < 0.001). Post hoc tests showed that both CO (*P* = 0.004) and RO (*P* = 0.013) groups had significantly lower total fetal weight than the DW group, with no difference between CO and RO groups (*P* = 0.884, Fig. 1C). Similarly, average fetal weight was significantly reduced in both CO and RO groups (*P* < 0.001 for both) compared with DW, while no difference was found between CO and RO groups (*P* = 0.188, Fig. 1D).

### Maternal, placental, and fetal CORT concentrations

**Figure 2:**
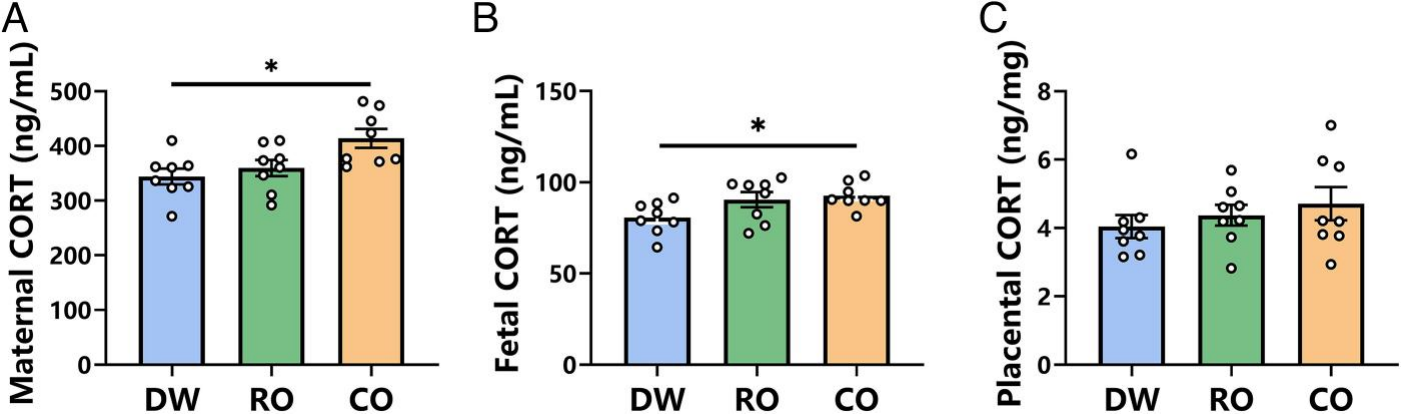
Effect of prenatal exposure to different odors on maternal, placental, and fetal corticosterone (CORT) concentration of Brandt's voles. (A) Maternal serum CORT level. (B) Fetal serum CORT level. (C) Placental CORT level. DW, distilled water exposure group, *n* = 8; RO, rabbit odor exposure group, *n* = 8; CO, cat odor exposure group, *n* = 8. Data are mean ± SEM. Significant differences are indicated: *P < 0.05.

The 1-way ANOVA results showed that prenatal exposure to different odors had a significant impact on CORT levels in both maternal (*F*_2, 21_ = 5.565, *P* = 0.011, Fig. 2A) and fetal (*F*_2, 21_ = 3.675, *P* = 0.043. Fig. 2B) serum of Brandt's vole but did not affect CORT levels in the placenta (*F*_2, 21_ = 0.751, *P* = 0.484, Fig. 2C). Post hoc tests revealed that maternal serum CORT levels were significantly higher in the CO group than in the DW group (*P* = 0.012), with no significant differences between RO and DW (*P* = 0.762) or between RO and CO groups (*P* = 0.056, Fig. 2A). Similarly, fetal serum CORT was elevated in the CO group compared to DW (*P* = 0.030), while no significant differences were observed between RO and DW (*P* = 0.118) or between RO and CO groups (*P* = 0.887, Fig. 2B).

### Placental 11β-HSD1, 11β-HSD2, and P-gp expression

**Figure 3:**
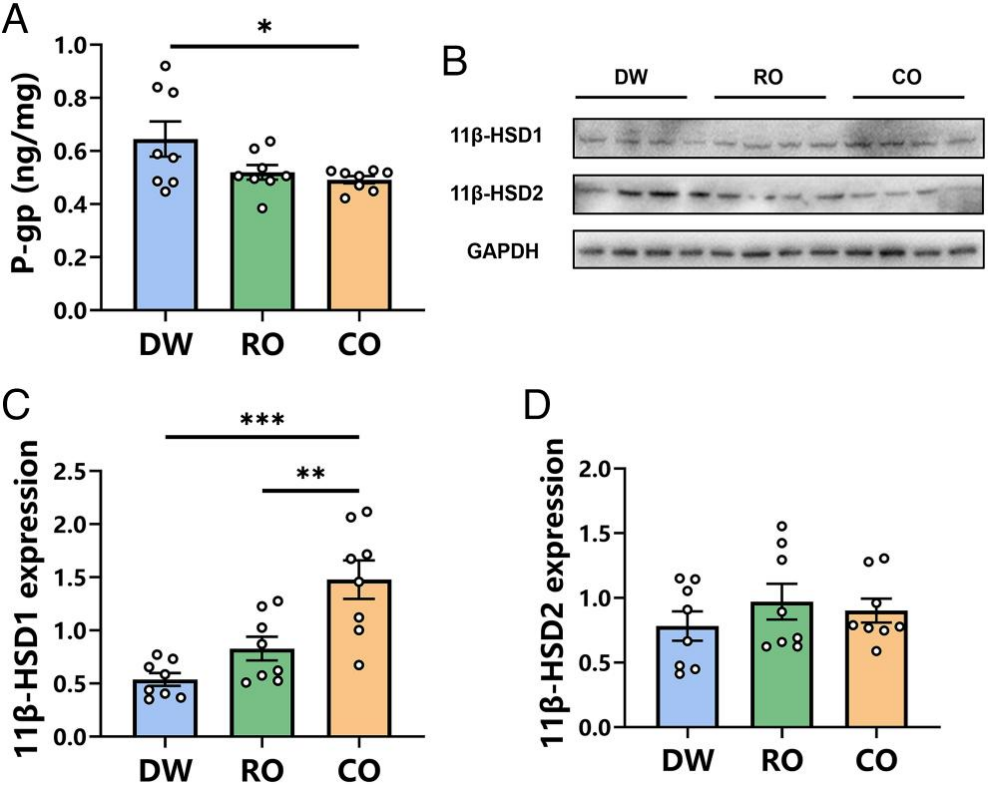
Effect of prenatal exposure to different odors on placental P-gp, 11β-HSD1, and 11β-HSD2 expression of Brandt's voles. (A) Placental P-glycoprotein (P-gp) expression. (B) Gel electrophoretic band pattern. (C) Placental 11β-hydroxysteroid dehydrogenase 1 (11β-HSD1) expression. (D) Placental 11β-hydroxysteroid dehydrogenase 2 (11β-HSD2) expression. DW, distilled water exposure group, *n* = 8; RO, rabbit odor exposure group, *n* = 8; CO, cat odor exposure group, *n* = 8. Data are mean ± SEM. Significant differences are indicated: *P < 0.05, **P < 0.01, ***P < 0.001.

The one-way ANOVA revealed that prenatal exposure to different odors significantly affected placental P-gp levels in Brandt's voles (*F*_2, 21_ = 3.775, *P* = 0.040, Fig. 3A). Post hoc tests indicated that placental P-gp levels in the CO group were significantly lower than those in the DW group (*P* = 0.044, Fig. 3A). There was no significant difference in placental P-gp levels of RO voles compared to DW (*P* = 0.113) or CO voles (*P* = 0.884, Fig. 3A).

Prenatal exposure to different odors significantly impacted the expression of 11β-HSD1 protein in the placenta of Brandt's voles (*F*_2, 21_ = 14.160, *P* < 0.001, Fig. 3B,C), but it did not affect the expression of 11β-HSD2 protein (*F*_2, 21_ = 0.668, *P* = 0.523, Fig. 3B,D). *Post hoc* tests revealed that placental 11β-HSD1 expression in the CO group was significantly higher than in both the RO group (*P* = 0.005) and the DW group (*P* < 0.001, Fig. 3B,C). There was no significant difference in placental 11β-HSD1 expression of RO voles compared to DW (*P* = 0.265, Fig. 3B,C). The corresponding Western blot images for all analyzed placental tissues are shown in Supplementary Fig. S1.

### CRH and GR expression in the fetal brain

**Figure 4:**
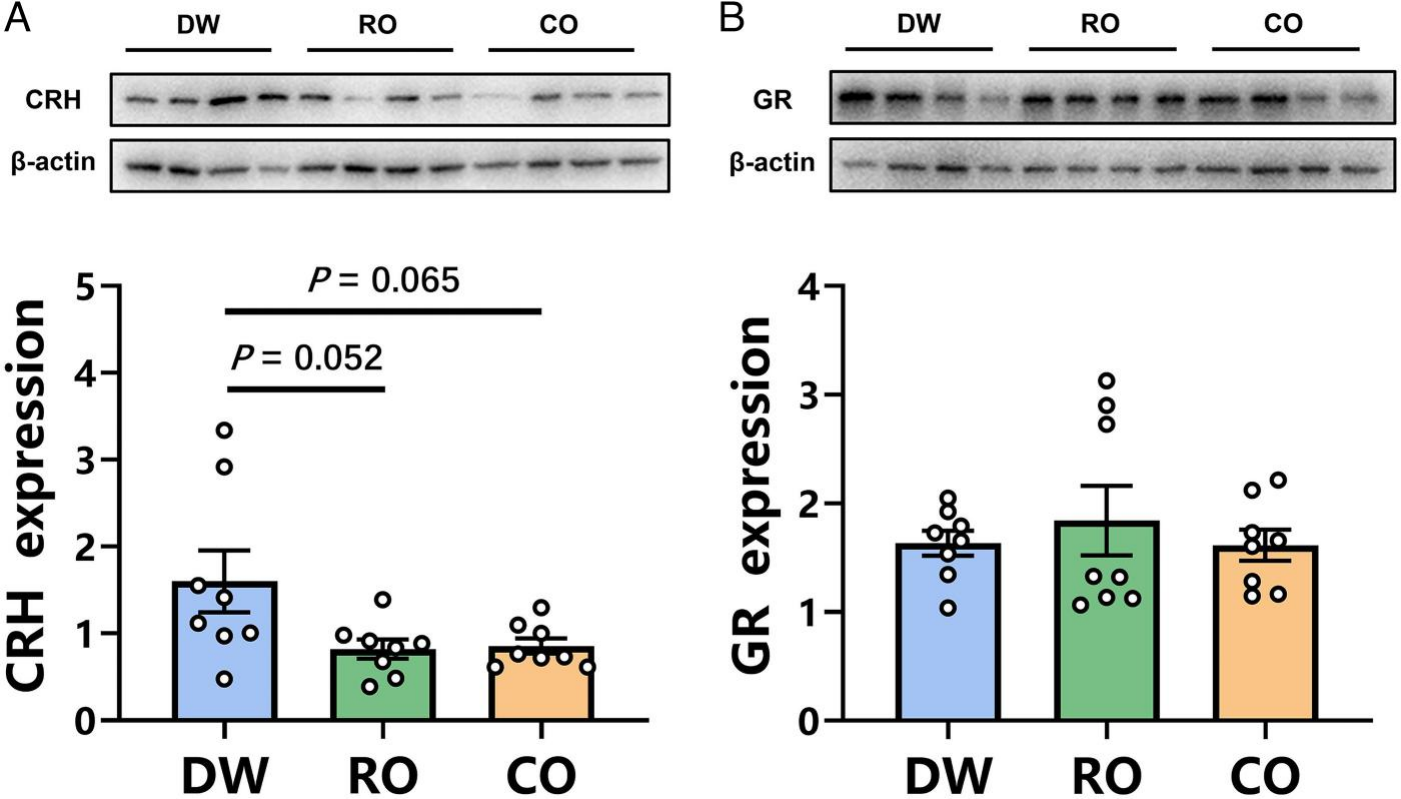
Effect of prenatal exposure to different odors on the expression of hypothalamic corticotropin-releasing hormone (CRH, A) and hippocampal glucocorticoid receptor (GR, B) of the fetus in Brandt's voles. DW, distilled water exposure group, *n* = 8; RO, rabbit odor exposure group, *n* = 8; CO, cat odor exposure group, *n* = 8. Data are mean ± SEM. Significant differences are indicated: *P < 0.05.

The one-way ANOVA results showed that prenatal exposure to different odors significantly affected CRH expression in the fetal hypothalamus of Brandt's vole (*F*_2, 21_ = 3.997, *P* = 0.034, Fig. 4A) but did not affect GR expression in the hippocampus (*F*_2, 21_ = 0.347, *P* = 0.711, Fig. 4B). Post hoc tests revealed that CRH expression in the fetal hypothalamus tended to decrease in both the RO group (*P* = 0.052) and the CO group (*P* = 0.065) compared to the DW group (Fig. 4A). There was no significant difference in fetal CRH expression of RO voles compared to CO voles (*P* = 0.993, Fig. 4A). The corresponding Western blot images for all analyzed fetal hypothalamic and hippocampal tissues are shown in Supplementary Figs. S2 and S3.

### Correlations between CORT concentrations and relative protein expression

**Figure 5:**
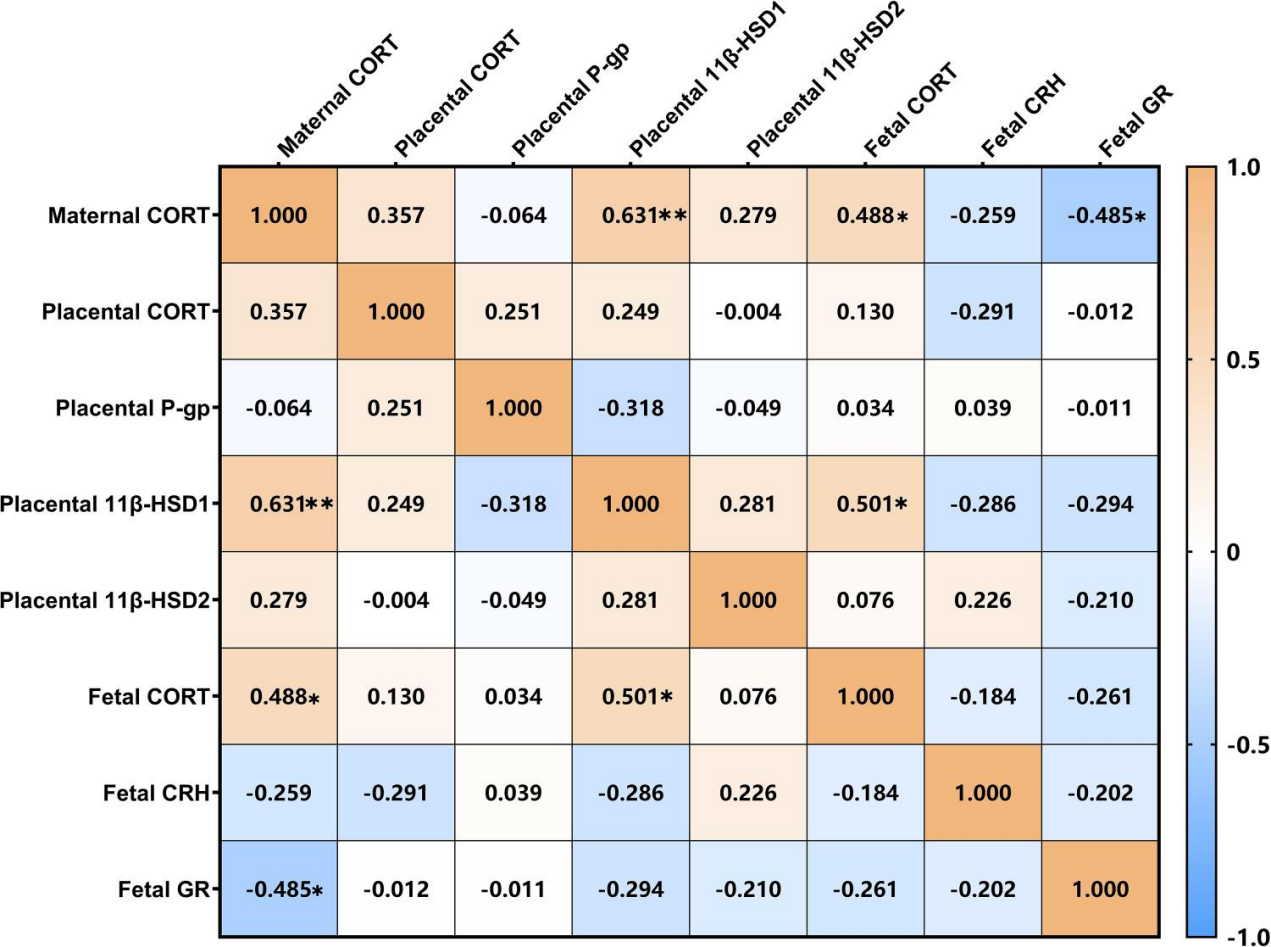
Correlation heat map of maternal, placental, and fetal variables. The matrix illustrates Pearson correlation coefficients between maternal corticosterone (CORT), placental CORT, placental P-glycoprotein (P-gp), placental 11β-hydroxysteroid dehydrogenase 1 (11β-HSD1), placental 11β-hydroxysteroid dehydrogenase 2 (11β-HSD2), fetal CORT, fetal corticotropin-releasing hormone (CRH), and fetal glucocorticoid receptor (GR). Positive correlations are represented by orange shades, while negative correlations are depicted in blue shades, with color intensity corresponding to the strength of the correlation. Correlation coefficients (*r*) are annotated within the cells. Significant differences are indicated: *P < 0.05, **P < 0.01.

As shown in Fig. 5, maternal CORT level had significant positive correlations with placenta 11β-HSD1 expression (*r* = 0.631, *P* = 0.001) and fetal CORT level (*r* = 0.488, *P* = 0.016) but had a significant negative correlation with fetal GR expression (*r* = −0.485, *P* = 0.016). In addition, a significant positive correlation was observed between fetal CORT and placental 11β-HSD1 (*r* = 0.501, *P* = 0.013).

## Discussion

This study investigated the effects of prenatal exposure to predator odor on placental GC metabolic function and fetal development in Brandt's voles. Our findings revealed that such exposure significantly reduced fetal weight and altered the expression of key GC-regulatory proteins in placenta, specifically 11β-HSD1 and P-gp. These molecular changes were accompanied by elevated CORT levels in both maternal and fetal serum, suggesting that predator-induced stress may disrupt placental GC metabolism, thereby increasing fetal exposure to maternal GCs and potentially impairing fetal development.

One of the most important findings of this study was that the total and average weight of the fetus decreased significantly after the mother was exposed to predator odors. This result is consistent with the hypothesis that prenatal stress can lead to intrauterine growth restriction (IUGR), a condition associated with various adverse outcomes in mammals (Resnik 2002). In our study, fetal weight loss in voles exposed to predator odors could be attributed to disruption of placental barrier function, which usually regulates the transfer of nutrients and hormones between mother and fetus. Damage to this barrier can lead to nutritional deficiencies in the fetus and changes in the hormonal environment, which are essential for fetal growth and development (Zhu et al. 2019). However, it should be noted that although IUGR is generally associated with negative outcomes, some low-birth-weight organisms may recover from potential impacts through catch-up growth (Hales and Ozanne 2003; Alexeev et al. 2015). In this light, it is possible that the birth weight deficits detected in this study are temporary and do not necessarily translate into poorer long-term outcomes. In contrast, recent rodent meta-analyses on prenatal stress suggest that catch-up growth often fails to occur, resulting in sustained reductions in body weight (Bermejo et al. 2022). In this study, we did not track offspring beyond birth, so it remains unclear whether lower birth weights persist or are offset by compensatory growth. Further longitudinal research with repeated measures of growth and survival would more accurately determine whether the reduced birth weight observed here reflects a lasting developmental constraint or a flexible physiological response that allows subsequent catch-up growth.

To further understand how these effects arise, we examined the interplay between placental CORT levels and maternal/fetal serum CORT concentrations. While placental CORT levels remained unchanged, both maternal and fetal serum CORT levels were significantly elevated in predator odor-exposed groups, likely reflecting activation of the HPA axis activation (Harris and Carr 2016). The discrepancy between serum and placental CORT levels suggests that the placental GC metabolism function may have been overwhelmed or impaired, potentially due to altered expression of key regulatory enzymes such as 11β-HSD1 and 1β-HSD2. These enzymes are critical in regulating fetal exposure to GCs (Benediktsson et al. 1997; Sun and Myatt 2003). While 11β-HSD2 typically protects the fetus by converting active GCs into their inactive forms, thereby serving as a barrier against excessive GC exposure (Chapman et al. 2013; Cottrell et al. 2014), our study observed no significant changes in its expression in response to predator odor exposure. Instead, we found an upregulation of 11β-HSD1, regenerating active GCs from their inactive forms, potentially increasing the local concentration of active GCs within the placenta. This could exacerbate the effects of elevated maternal CORT levels by enhancing fetal exposure to these hormones, thus contributing to the observed fetal growth restrictions (St-Pierre et al. 2016; Miranda and Sousa 2018). Previous studies have primarily focused on the downregulation of 11β-HSD2 as a response to prenatal stress. For example, upregulation of 11β-HSD2 may protect the fetus against stress-induced elevations of maternal CORT, but exposure to chronic stress greatly diminishes this protection in rats (Welberg et al. 2005). In human studies, mothers who reported prenatal anxiety and depression had increased methylation of 11β-HSD2 in their placentas, which was positively correlated with infant neurobehavioral disorders (Conradt et al. 2013; Appleton et al. 2015). While much attention has been paid to 11β-HSD2 downregulation in stress research, our findings suggest that upregulation of 11β-HSD1, even in the absence of 11β-HSD2 suppression, may represent an alternative mechanism through which maternal stress influences fetal development.

Alongside these enzyme alterations, the reduced expression of P-gp observed in predator odor-exposed voles further underscores the potential compromise of the placental barrier. P-gp is a well-known transporter protein that plays a crucial role in maintaining placental barrier integrity by limiting the transfer of harmful substances, including GCs, from the mother to the fetus (Harris and Seckl 2011). Our finding of reduced P-gp expression in response to predator odor exposure is concerning, as it suggests a weakened placental barrier that could allow for greater maternal–fetal transfer of CORT and other stress-related hormones. This reduction in P-gp expression aligns with previous research showing that various stressors, including maternal malnutrition and environmental toxins, can compromise P-gp expression, leading to increased fetal exposure to xenobiotics and GCs (Ceckova-Novotna et al. 2006; Seckl and Holmes 2007). The differential expression of 11β-HSD1 and P-gp suggests that the combination of reduced P-gp expression and increased 11β-HSD1 activity may work synergistically to elevate fetal GC exposure, thereby impacting fetal development. Although 11β-HSD1 is typically involved in the local regeneration of active GCs, its upregulation may respond to maternal stress and exacerbate fetal exposure to GCs (Maeyama et al. 2015). Conversely, reduced P-gp expression may indicate a diminished efflux capacity of these hormones, thereby impairing the protective barrier function of the placenta. These changes are critical because they may help observe changes in fetal growth patterns and have broader implications for programming fetal stress responses.

Interestingly, although our results revealed no change in hippocampal GR levels, the downward trend in hypothalamic CRH expression suggests that prenatal stress may primarily affect the upstream regulatory elements of the HPA axis rather than altering receptor sensitivity. This could imply that the fetus experiences a blunted or altered HPA axis response, potentially due to feedback inhibition caused by elevated maternal CORT levels. These findings align with research suggesting that prenatal stress can differentially affect various components of the HPA axis. For example, Kapoor et al. (2008) have summarized that prenatal stress led to increased CRH expression but did not always correspond with changes in GR expression, leading to dysregulated stress responses in offspring. Similarly, studies by Welberg et al. (2000) have shown that prenatal glucocorticoid exposure can lead to long-lasting changes in CRH expression and HPA axis activity, which are not always accompanied by changes in GR expression. Overexposure to CORT may have impaired the function of the fetal HPA axis (Harris 2020), leading to a mismatch between hypothalamic CRH expression and serum CORT levels. The long-term consequences of altered CRH expression in the fetal brain, especially considering the potential for transgenerational effects of prenatal stress, warrant further investigation.

Our initial interpretation emphasized the detrimental effects of maternal stress, particularly when elevated GCs compromise fetal growth or endocrine function. However, it is essential to acknowledge scenarios in which maternal stress transmission can be adaptive (Marshall and Uller 2007). In predator-rich environments, maternal stress signals may prepare offspring for heightened environmental challenges by enhancing their ability to respond effectively to threatening situations later in life. Indeed, our previous study also found that offspring exposed to maternal predation stress exhibited stronger behavioral and physiological responses to acute predation risk as they matured (Gu et al. 2023). Other studies have similarly shown that offspring exposed to prenatal stress can develop heightened vigilance, bolder anti-predator behavior, or altered stress physiology, all of which may boost survival probabilities (St-Cyr et al. 2017, 2018; Sievert et al. 2020). However, if the postnatal environment does not align with the predicted risks (e.g., if actual predation risk is low), offspring may face the metabolic or growth costs of a mismatched phenotype (Marshall and Uller 2007; Sheriff and Love 2013; St-Cyr and McGowan 2018). Therefore, the fitness value of maternal stress signals is context-dependent and species-specific.

Moreover, our results highlight that even non-predator odors, such as rabbit urine, can elicit considerable maternal stress responses in Brandt's voles. The changes observed in the RO group, including reduced fetal weight and decreased CRH protein expression, suggest that even non-predator odors can elicit significant maternal stress responses. Previous research has shown that unpleasant odors can impair an individual's mood and induce stress responses, even when the odorants are non-toxic (Hirasawa et al. 2019). The novelty of rabbit urine odor may have been unpleasant to voles, sufficiently activating the maternal HPA axis and resulting in increased CORT levels that affected fetal growth and neuroendocrine regulation. However, unlike the cat odor exposure group, rabbit odor did not appear to affect the expression of placental barrier proteins such as 11β-HSD1 and P-gp, indicating that the placental barrier remained functionally intact. This difference underscores the specificity of stress responses induced by predator versus non-predator odors. Predator odors may exert a more potent effect, potentially compromising placental function, whereas the stress response to a novel but non-predator odor seems to impact fetal development without altering placental GC metabolism. Additionally, Sánchez-González et al. (2017) demonstrated that wood mice (*Apodemus sylvaticus*) exhibited a significant rise in fecal corticosterone metabolites (FCM) in plots containing low, medium, and high concentrations of predator odor. Meanwhile, hormonal secretion increased with increasing concentration of the fecal odor. These findings suggest that the intensity of a stressor may also play a critical role in determining maternal and fetal outcomes. Natural, undiluted urine could provide a more realistic ecological stimulus and may induce greater maternal stress, thereby better distinguishing the different effects elicited by predator and non-predator odors.

Several limitations should be acknowledged. First, our interpretation of placental dysfunction is based solely on molecular markers related to GC metabolism and transport, without direct histological or ultrastructural evidence of changes in placental barrier integrity. Future studies should incorporate structural assessments to validate and extend our current findings. Second, although we observed significant changes in fetal weight and placental protein expression, the absence of postnatal follow-up limits our ability to assess the long-term developmental outcomes of these prenatal effects. Finally, because this study focused exclusively on GC-related pathways, other potential mechanisms, such as placental vascular remodeling, altered nutrient transport, and immune activation (Reynolds et al. 2013), were not investigated. Addressing these limitations in future work will be essential for a more comprehensive understanding of how ecological stressors shape fetal development.

In conclusion, this study demonstrates that prenatal exposure to predator odor alters placental glucocorticoid metabolism and potentially impairs fetal growth in Brandt's voles. These effects appear to be mediated by elevated maternal CORT levels and dysregulated expression of placental enzymes and transporters, particularly 11β-HSD1 and P-gp. Although fetal weight reductions were also observed in response to non-predator odor, only predator odor exposure induced changes in placental GC-related proteins, suggesting a stressor-specific effect on placental endocrine function. Depending on the ecological context, these findings highlight the importance of placental GC regulation as a mediator of prenatal environmental effects, with potential implications for adaptive or maladaptive developmental programming.

## Supplementary Material

zoaf043_Supplementary_Data
