# Supplementary material for: Prenatal exposure to predator odor alters placental glucocorticoid metabolism and affects fetal growth in Brandt's voles (Lasiopodomys brandtii)
Source: Curr Zool. 2025 Jul 28;72(2):273–82. doi: 10.1093/cz/zoaf043 (PMC13202339; doi:10.1093/cz/zoaf043)
Supplement: zoaf043_Supplementary_Data [file zoaf043_supplementary_data.docx]

**Supplementary materials for:**

Prenatal exposure to predator odor alters placental glucocorticoid metabolism and affects fetal growth in Brandt’s voles (*Lasiopodomys brandtii*)

**Running title:** Effects of prenatal predator stress on fetus

**Authors and contact information:** Chen Gu, Yang Yu, Yuqing Zhang, Fengping Yang, Shengmei Yang, Baofa Yin*, and Wanhong Wei*

Department of Animal Behavior, College of Bioscience and Biotechnology, Yangzhou University, Yangzhou, 225009, China

*Corresponding Authors: Baofa Yin, Wanhong Wei

Email: bfyin@yzu.edu.cn; whwei@yzu.edu.cn.


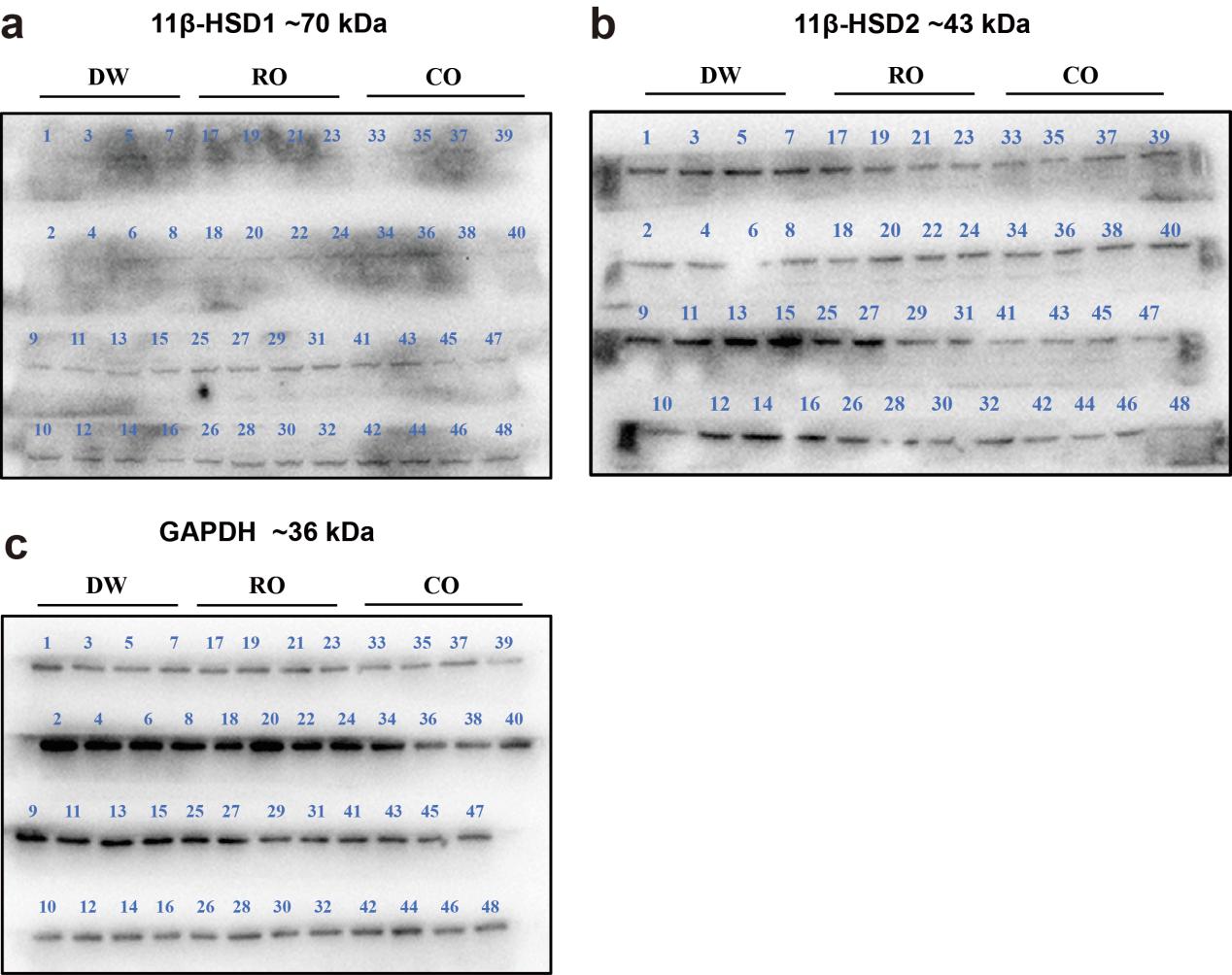


**Figure S1**. Western blot images of placental 11β-hydroxysteroid dehydrogenase type 1 (11β-HSD1, ~70 kDa, a), 11β-hydroxysteroid dehydrogenase type 2 (11β-HSD2, ~43 kDa, b), and GAPDH (~36 kDa, c) in Brandt’s voles. All 48 individual placental samples used in the analysis are presented. Samples 1–16 correspond to the distilled water (DW) group, 17–32 to the rabbit odor (RO) group, and 33–48 to the cat odor (CO) group. Each sample number represents a placental tissue sample, and every pair of samples with numerically consecutive IDs (e.g., 1 and 2, 3 and 4, etc.) was derived from the same mother. GAPDH was used as an internal loading control.


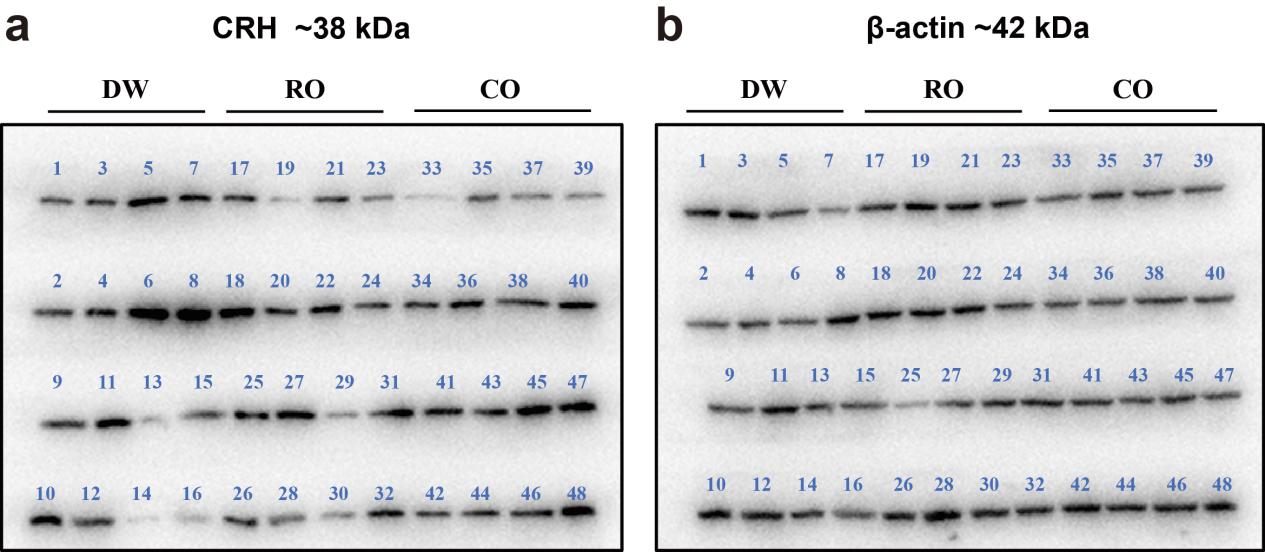


**Figure S2**. Western blot images of corticotropin-releasing hormone (CRH, ~38 kDa, a) and β-actin (~42 kDa, b) expression in the fetal hypothalamus of Brandt’s voles. All 48 individual fetal brain samples used in the analysis are presented. Samples 1–16 correspond to the distilled water (DW) group, 17–32 to the rabbit odor (RO) group, and 33–48 to the cat odor (CO) group. Each sample number represents a fetal individual, and every pair of samples with numerically consecutive IDs (e.g., 1 and 2, 3 and 4, etc.) was derived from the same mother. β-actin was used as an internal loading control.


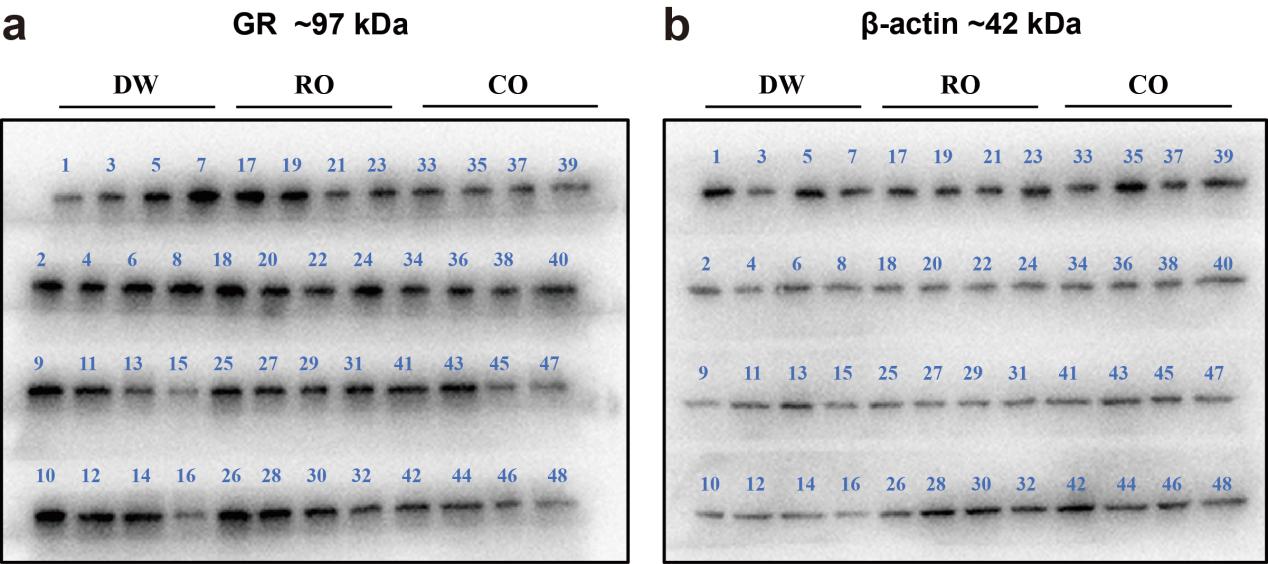


**Figure S3**. Western blot images of glucocorticoid receptor (GR，~38 kDa, a) and β-actin (~42 kDa, b) expression in the fetal hippocampus of Brandt’s voles. All 48 individual fetal brain samples used in the analysis are presented. Samples 1–16 correspond to the distilled water (DW) group, 17–32 to the rabbit odor (RO) group, and 33–48 to the cat odor (CO) group. Each sample number represents a fetal individual, and every pair of samples with numerically consecutive IDs (e.g., 1 and 2, 3 and 4, etc.) was derived from the same mother. β-actin was used as an internal loading control.
